# Cognitive impairment in Parkinson disease correlates with loss of cholinergic axon projections to cortex in an autopsy study

**DOI:** 10.1002/alz.71868

**Published:** 2026-10-06

**Authors:** Rebecca Miller, Jennifer O'Shea, Laura M. Rosok, Sydney Lim, Erin E. Franklin, Richard J. Perrin, Helen Hwang, Dhruva Dhavale, Joel Perlmutter, Meghan C. Campbell, Paul Kotzbauer

**Affiliations:** ^1^ Department of Neurology Washington University School of Medicine St. Louis Missouri USA; ^2^ Hope Center for Neurological Disorders Washington University School of Medicine St. Louis Missouri USA; ^3^ Department of Pathology and Immunology Washington University School of Medicine St. Louis Missouri USA; ^4^ Knight Alzheimer Disease Research Center Washington University School of Medicine St. Louis Missouri USA; ^5^ Department of Radiology Washington University School of Medicine St. Louis Missouri USA; ^6^ Department of Neuroscience Washington University School of Medicine St. Louis Missouri USA; ^7^ Program in Occupational Therapy Washington University School of Medicine St. Louis Missouri USA; ^8^ Program in Physical Therapy Washington University School of Medicine St. Louis Missouri USA; ^9^ Department of Developmental Biology Washington University School of Medicine St. Louis Missouri USA

**Keywords:** choline acetyltransferase, cholinergic neurons, Lewy body dementia, Parkinson's disease

## Abstract

**INTRODUCTION:**

People with Parkinson's disease (PD) frequently develop dementia. We investigated the relationship between cognitive impairment and the loss of cholinergic axon projections from the nucleus basalis of Meynert to neocortical regions in PD.

**METHODS:**

Levels of choline acetyltransferase (ChAT), the enzyme required for acetylcholine synthesis, were measured with a sandwich enzyme‐linked immunosorbent assay (ELISA) in eight brain regions from 37 PD autopsy cases. Analyses focused on relationships between measures of global cognitive impairment and average ChAT levels in four neocortical regions.

**RESULTS:**

Mean neocortical ChAT levels positively correlated with the Mini‐Mental State Examination and a global cognition z‐score obtained by averaging performance in multiple cognitive domains. Mean neocortical ChAT levels also showed a trending inverse correlation with the Clinical Dementia Rating sum of boxes.

**DISCUSSION:**

These results indicate a role for the loss of cholinergic projections to neocortical regions in the development of cognitive impairment in PD.

## Highlights

- We developed a sandwich enzyme‐linked immunosorbent assay (ELISA) to measure choline acetyltransferase (ChAT) levels.
- Cholinergic axon projections to neocortical regions are reduced in Parkinson disease.
- Reduced cholinergic projections to neocortex correlates with cognitive impairment.

## 1 BACKGROUND

Parkinson's disease (PD) is the second most common neurodegenerative disorder. Its motor features include tremor, bradykinesia, rigidity, and impaired postural reflexes.^1^ In addition to these motor symptoms, the majority of PD patients also develop non‐motor symptoms,^1^, ^2^ with up to 80% developing dementia.^3^, ^4^, ^5^ The timing of dementia onset relative to motor symptom onset, however, is highly variable. The term Lewy body dementia (LBD) encompasses the spectrum of dementia onset, which can occur less than one year after the onset of motor symptoms (often referred to as dementia with Lewy bodies [DLB]), or up to 20 years or more after the onset of motor symptoms (PD with dementia [PDD]).

The progressive loss of dopaminergic neurons in substantia nigra pars compacta underlies many of the motor symptoms in PD. Other subcortical neuronal populations are also affected in PD and may contribute to both motor and non‐motor symptoms. For example, dysfunction of cholinergic neurons is implicated in motor and non‐motor symptoms, including gait disorders, postural instability, tremor, rigidity, rapid eye movement behavior disorder, olfaction impairment, and cognitive deficits.^6^, ^7^, ^8^, ^9^, ^10^, ^11^ Studies have suggested that cholinergic system dysfunction occurs early in PD, and it may occur as early as the loss of dopaminergic neurons.^8^, ^11^, ^12^, ^13^

The cholinergic basal forebrain neurons arise from the medial septum/vertical limb of the diagonal band (Ch1‐Ch2), and the nucleus basalis of Meynert (NBM) (Ch4) that innervate the hippocampus (Ch1 and Ch2) and neocortex and amygdala (Ch4).^5^, ^7^, ^11^, ^14^, ^15^ Lewy bodies and loss of cholinergic neurons in NBM (Ch4) are a common pathologic finding in PD. Magnetic resonance imaging (MRI) ‐based volumetric analyses of the substantia innominata (SI), a region that contains NBM as segmented in the MRI scans, indicate lower SI volume in PD compared to aged controls, and greater atrophy in PDD compared to PD without dementia.^6^, ^13^, ^16^, ^17^, ^18^, ^19^ In addition, loss of gray matter volume in the NBM, as measured by MRI, precedes and predicts the onset of cognitive impairment in PD.^4^, ^20^, ^21^ Positron emission tomography (PET) imaging with a vesicular acetylcholine transporter (VAChT) tracer demonstrates correlations between loss of cholinergic innervation and cognitive impairment in multiple brain regions.^5^

Studies in *post mortem* tissue can be used to further investigate the loss of cholinergic projections to the cortex and other brain regions in PD, and the degree to which loss of cholinergic projections contributes to both global cognitive impairment and specific cognitive phenotypes in PD. Several markers of cholinergic neurons have been utilized to examine dysfunction of the cholinergic system in PD *post mortem* tissue, including the synthetic enzyme ChAT, the transporter VAChT, the metabolizing enzyme acetylcholinesterase (AChE) and the neurotrophin receptor p75^NTR^.^22^ In *post mortem* brain tissue using a radiometric enzyme assay, cortical ChAT activity has been shown to be significantly reduced in PD compared to controls, with DLB and PDD having reduced ChAT activity compared to PD.^23^, ^24^, ^25^, ^26^, ^27^, ^28^ ChAT immunostaining in *post mortem* tissue sections has also demonstrated substantial loss of cholinergic neurons in NBM, as well as loss of cholinergic axons in the capsular branch of the lateral cholinergic pathway adjacent to NBM.^23^ Immunostaining for p75^NTR^ has also shown loss of cholinergic neurons in NBM, accompanied by reduced ChAT and AChE containing axons in temporal cortex.^22^ To pursue higher throughput measurements of cholinergic projections in larger numbers of brain regions and autopsy cases, we utilized a sensitive sandwich ELISA to quantify the levels of ChAT in *post mortem* tissue. We applied this sandwich ELISA approach to *post mortem* tissue obtained from eight brain regions collected from control, PD and LBD autopsy cases from a longitudinal observational study of PD. We focused our initial analysis on the relationship between mean neocortical ChAT levels and global cognitive impairment in PD.

**RESEARCH IN CONTEXT**

1. **Systematic review**: The authors reviewed the literature using traditional sources (e.g., PubMed). The relationship between cognitive impairment in Parkinson's disease (PD) and the loss of cholinergic axon projections from the nucleus basalis of Meynert to cortex has been investigated by measuring choline acetyltransferase (ChAT) enzyme activity in *post mortem* tissue studies as well as by magnetic resonance imaging and positron emission tomography imaging in clinical studies. These previous studies are appropriately cited.
2. **Interpretation**: Our findings, utilizing a sandwich enzyme‐linked immunosorbent assay (ELISA) to measure ChAT in *post mortem* tissue samples, demonstrate a relationship between measures of global cognitive impairment and loss of cholinergic axon projections to neocortical regions in PD.
3. **Future directions**: Our findings complement the results of imaging studies and previous *post mortem* tissue studies. Application of the ChAT sandwich ELISA in future studies with larger numbers of autopsy cases enables further investigation of the role of cholinergic degeneration in global cognitive impairment, as well as specific cognitive phenotypes in PD.

## 2 METHODS

### 2.1 Standard protocol approvals and patient consents

This research was conducted in accordance with the Declaration of Helsinki. The Human Research Protection Office at Washington University in Saint Louis approved this study. Written informed consent to perform a brain autopsy was obtained from all participants. After death, the immediate next‐of‐kin were contacted and confirmed consent for brain removal and retention of brain tissue for research purposes.

### 2.2 Study participants

The cohort included 29 PD participants with dementia (LBD), eight PD participants without dementia, and six healthy age‐matched neurologically normal control participants. All PD participants and one control participant were recruited through the Movement Disorders Center (MDC), and five control participants were recruited through the Knight Alzheimer Disease Research Center (ADRC), as described previously.^29^, ^30^ Both centers are located at Washington University in Saint Louis. The five aged controls from the ADRC were included posthumously based on clinical and neuropathologic criteria. *Post mortem* brain tissue was collected between April 2008 and May 2018 and evaluated by immunohistochemical (IHC) staining and neuropathological staging as previously described.^31^, ^32^, ^33^ Inclusion criteria for the study were: (a) PD participants with dementia who had a clinical diagnosis of idiopathic PD based on the United Kingdom Parkinson Disease Society Brain Bank diagnostic criteria^34^ and either Clinical Dementia Rating (CDR global score)^35^ ≥1 or a clinical diagnosis of dementia; (b) PD participants without dementia who had a clinical diagnosis of idiopathic PD based on the United Kingdom Parkinson Disease Society Brain Bank diagnostic criteria and CDR ≤ 0.5 or did not have a clinical diagnosis of dementia; and (c) control participants with CDR ≤ 0.5, Braak Stage 0 LB pathology, Braak stage 0‐B Aβ pathology^31^ and Braak stage I‐III tau pathology.^36^ The median age for control (four female and two males) was 85, PD (five male and three female) was 73, and LBD (23 males and 6 females) was 78 (Table 1).

**TABLE 1:** Demographic and clinical information of the autopsied cases.

Parameter	Control	PD	LBD
Participants, *N*	6	8	29
Age at death, *y*	85 (70‐101)	73 (65‐87)	78 (65‐93)
Male/female, *N*	2/4	5/3	23/6
Age at diagnosis, *y*	NA	60 (54‐71)	62 (49‐85)
UPDRS‐III score (OFF medications)	NA	37 (25‐50)	40 (32‐74)
Education, *y*	NA	15 (14‐20)	15 (12‐20)
PD duration, *y*	NA	10 (7‐25)	16 (6‐25)
CDR sum of boxes (max. score = 18)	NA	1 (0.5‐2.5)	7 (2‐18)
Interval between last CDR and death (*y*)	NA	0 (0‐3)	2 (0‐7)
MMSE (max. score = 30)	NA	28 (25‐30)	20 (3‐28)
Interval between last Cog‐Z and death (*y*)	NA	2 (0‐3)	2 (0‐7)
Cog‐Z	NA	−0.38 (−0.72−1.16)	−0.11 (−2.53−0.10)
Interval between last MMSE and death (*y*)	NA	2 (0‐3)	1 (0‐6)
*Post mortem* interval (h)	8.25 (6‐16)	12 (5.5‐69)	11 (2.4‐52)

Group differences were tested using independent‐samples *t*‐tests for continuous variables and chi‐square tests for categorical variables. Levene's test indicated equal variances for all *t*‐test comparisons (*p* > 0.05). No significant differences were observed between groups for any of the listed variables (all *p*’s > 0.05). Abbreviations: CDR, Clinical Dementia Rating; Cog‐Z, global cognition z‐score; LBD, Lewy body disease; MMSE, Mini‐Mental State Examination; NA, not applicable; PD, Parkinson's Disease; UPDRS, Unified Parkinson Disease Rating Scale; y, years.

### 2.3 Motor assessments

MDC clinicians evaluated motor symptoms using the Unified Parkinson Disease Rating Scale motor subscale III (UPDRS‐III).^37^ Evaluations were performed OFF anti‐parkinsonian medications.

### 2.4 Dementia ratings

Dementia was assessed using the CDR^35^ scale by certified raters. The CDR evaluates whether cognitive dysfunction is sufficiently severe to impair activities of daily living. PD participants were recruited into the longitudinal study regardless of cognitive status or the timing of cognitive decline relative to the onset of motor symptoms. Prior to death, all PD participants with dementia had a CDR ≥ 1 or clinical diagnosis of dementia determined by a neurologist, and PD participants without dementia had a CDR ≤ 0.5 and did not have a clinical diagnosis of dementia at death, in accordance with criteria for dementia in PD.^38^ The CDR sum of boxes, a related metric to the global CDR with greater score variation (0–18), was used as a variable of interest in analyses. Values were taken from assessments closest to the time of death.

### 2.5 Cognitive testing

Global cognitive function was assessed using the Mini‐Mental State Examination (MMSE), a widely used cognitive screening tool, with scores ranging from 0 to 30. In addition, a second measure of global cognition, referred to here as global cognition z‐score (Cog‐Z), was calculated by averaging performance across multiple cognitive domains: attention (Digit span; Digit Symbol^39^), memory (California Verbal Learning Test‐ II, short form^40^; Logical Memory^41^), language (Boston Naming test^42^), visuospatial function (Judgement of Line Orientation^43^; Spatial Relations Test^44^), and executive function (Trail Making Test^45^; DKEFS Verbal Fluency‐Switching and Color‐Word Interference^46^). Raw scores were converted to z‐scores based on normative data and then averaged to compute a global cognitive z‐score (Cog‐Z). Inability to complete a cognitive test, due to cognitive deficits, was imputed as *z* = −3 to indicate severe impairment. As with the CDR, MMSE and Cog‐Z scores closest to the time of death were used in analyses. Since five of the control participants were from the Knight ADRC and were not enrolled in our study, we have CDR scores but not MMSE and Cog‐Z scores.

### 2.6 Tissue collection, dissection, and extraction

At autopsy, the left hemibrain was fixed for 1 to 2 weeks in 10% buffered formal saline prior to block taking and embedding.^47^ To preserve frozen brain tissue, 1 cm thick coronal slices were made through the entire right hemisphere, placed between pre‐cooled Teflon‐coated aluminum plates, and frozen by immersion in liquid nitrogen vapor in an insulated (MVA 230, Thermo Fisher Scientific, St. Louis, MO) storage vessel. Frozen slices were placed into freezer grade (air‐tight) Ziploc bags and stored at ‐80°C. Frozen tissue dissection and extraction were previously described.^30^ Briefly, the eight regions of middle frontal gyrus (MFG), anterior cingulate gyrus (ACG), caudate, inferior parietal lobule (IPL), visual association cortex (VAC), precuneus, hippocampus, and amygdala were dissected from the right hemibrain. Using a scalpel, gray matter from the caudate nucleus and cortical regions was selectively dissected from the surrounding white matter while maintaining the brain tissue between ‐10°C and ‐15°C using dry ice. For hippocampus and amygdala regions, multiple 100 µm tissue sections were sectioned through the entire regions using a cryotome maintained at ‐15°C. For all regions, approximately 1 gram of tissue of cortical regions, or the entire amount of tissue from the other regions, was dissected and reduced to fragments of ∼1 mm^3^ or smaller and then distributed into aliquots of 50‐100 mg for subsequent extraction.

To prepare the extracts used for ChAT analysis, representative 100 mg aliquots from each region and case were sequentially homogenized in buffers of increasing detergent strength, with centrifugation at 100,000 × *g* after each homogenization step. Four different supernatants containing solubilized proteins were generated (high salt [HS]; 50 mM Tris‐HCl pH 7.5, 750 mM NaCl, 5 mM ethylenediaminetetraacetic acid [EDTA] plus 0.1× Sigma P2714 protease inhibitor cocktail), high salt + 1% Triton X‐100 (HS+T), radioimmunoprecipitation assay buffer, and 2% sodium dodecyl sulfate (SDS) in 50 mM Tris‐HCl, pH 7.4 buffer with 5 mg/L aprotinin and 50 mg/L leupeptin. It was determined by ELISA that soluble ChAT protein primarily partitioned into the HS fraction, but some was also detected in the HS+T fraction. Therefore, the HS and HS+T fractions were combined in equal parts, and these combined extracts were utilized for ChAT ELISA measurements. The ChAT protein would not have been denatured in HS and HS+T fractions since Triton X‐100 is a mild detergent. A subset of the samples used in this study was also utilized to produce the 2% SDS fraction for analysis of insoluble total alpha‐synuclein (asyn), phospho‐asyn (p129), Aβ, total tau and phospho‐tau (p395/404) from a previous study.^30^

### 2.7 Immunofluorescence staining

Immunofluorescence staining for ChAT was performed on fresh‐frozen *post mortem* brain tissue sections. Sections were cut on a cryostat to 16 µm and placed on glass slides. Then, sections were fixed in 4% paraformaldehyde (PFA) for 15 min, followed by 70% ethanol for 20 min. After the short fixation of PFA and ethanol, the sections were counterstained with 1× TrueBlack for 1 min to reduce autofluorescence, then washed with 1× phosphate‐buffered saline (PBS). The tissue was incubated overnight at 4°C with primary antibodies against ChAT: either goat anti‐ChAT (1:100, Sigma‐Aldrich AB144P) or goat anti‐ChAT (1:500, R&D Systems AF3447). Following washes, sections were incubated for 1 h at room temperature with an Alexa Fluor 488‐conjugated donkey anti‐goat secondary antibody (1:500, Jackson ImmunoResearch 705‐545‐147). After drying, sections were mounted using Fluoromount‐G. Images were captured on a Nikon Eclipse TE‐2000‐U Inverted Microscope, and image analysis was performed using Nikon Elements AR (version 5.42.02).

### 2.8 ChAT ELISA

Costar 96 well, clear, flat bottom, half area, high binding, polystyrene plates (Corning #3690) were used. The capture antibody (R&D Goat #AF3447) was diluted 1 µg/ml in 34 mM sodium bicarbonate, 16 mM sodium carbonate solution with 3 mM sodium azide. The capture antibody solution was added (50 µl/well) and incubated overnight at 4°C. Plates were washed 5× with 150 µl PBS with 0.05% Tween‐20/well using BioTek 405 TS plate washer. Plates were blocked with 150 µl of 2% bovine serum albumin (BSA) (Sigma‐Aldrich #A7906) in PBS at 37°C for 2 h. Prior to standard and sample addition, plates were washed 5× with 150 ml PBS with 0.05% Tween‐20/well using BioTek 405 TS plate washer. Samples were diluted in sample buffer containing 1% Triton X‐100, 0.1% BSA, 0.06% SDS, 5 mg/L aprotinin and 50 mg/L leupeptin in PBS. All brain region samples were diluted 1:5 in sample buffer except caudate samples. Caudate samples were diluted 1:3 in HS with 0.5% Triton X‐100 and then diluted 1:33.3 in sample buffer containing 1% Triton X‐100, 0.1% BSA, 0.06% SDS,17% HS with 0.5% Triton X‐100 (, 5 mg/L aprotinin and 50 mg/L leupeptin in PBS. Standard were prepared in sample buffer containing 1% Triton X‐100, 0.1% BSA, 0.06% SDS, 20% HS with 0.5% Triton X‐100, 5 mg/L aprotinin and 50 mg/L leupeptin in PBS. HS with 0.5% Triton X‐100 needed to be added to standard buffer (20%) and caudate sample buffer (17%; other 3% is from the caudate samples to total 20%) to be comparable to the sample conditions for the other regions that were diluted 5×, which would give a final concentration of 20% HS with 0.5% Triton X‐100. Standards were 10, 3.3, 1.1, 0.37, 0.12, 0.04, 0.014, 0 ng/ml. Standards and samples were added to the plate at 50 µl/well in duplicate wells and incubated overnight at 4°C with slow rotation. Plates were washed 5X with 150 ml PBS with 0.05% Tween‐20/well using a BioTek 405 TS plate washer. Detection antibody (CL3173; mouse monoclonal; Invitrogen #MA5‐31383) was diluted 1 µg/ml in PBS containing 0.05% BSA and 0.05% Tween‐20. Detection antibody solution was added at 50 µl/well and incubated at 37°C for 2 h. Plates were washed 5× with 150 ml PBS with 0.05% Tween‐20/well using a BioTek 405 TS plate washer. Peroxidase‐conjugated AffiniPure Goat Anti‐Mouse IgG, Fc gamma fragment specific (Jackson ImmunoResearch Cat # 115‐035‐071) was diluted 1:1000 in 1% BSA in PBS with 0.05% Tween‐20. The horseradish peroxidase (HRP) ‐antibody solution was added at 50 µl/well and incubated for 90 min at room temperature in the dark. The plate was washed as described previously. Fifty µl/well of 3,3′,5,5′‐Tetramethylbenzidine Liquid Substrate, Super Slow (Sigma # T5569) was rocked on the rotator until first reading. Readings were done on Synergy 2 (BioTek) at 650 nM at 9, 12, and 15 min. The time point with the highest ratio between the top concentration and 0 concentration (background) in the standard curve was chosen for data analysis. Four inter‐assay control extracts were run on all ELISA plates. The percent coefficient of variation (%CV), defined by standard deviation of a sample set divided by its mean multiplied by 100, was calculated to assess variation between sample sets. The %CV for the inter‐assay controls was 12% and the intra‐assay %CV was 10.8%.^48^

### 2.9 Statistical analyses

Data were analyzed using GraphPad Prism software, version 11 (Graph Pad Software, Inc., La Jolla, CA) and IBM SPSS Statistics, version 31.0.0.0 (117) (IBM Corp., Armonk, NY). Due to unequal group sizes, non‐parametric Mann‐Whitney *U* tests were utilized to compare group ChAT levels. Pearson correlations were conducted to examine associations between mean neocortical ChAT levels and measures of global cognitive function (i.e., CDR sum of boxes, MMSE, Cog‐Z). These analyses were followed by partial Pearson correlations controlling for time interval between data collection date and date of death, as well as years of education. All tests were two‐tailed and corrected for multiple comparisons with the Holm–Bonferroni method^49^ using a significance level of 0.05.

### 2.10 Standards

Recombinant ChAT protein used as a standard was produced by transient transfection of HEK293 cells in 10 cm dish (6.75 × 10^6^ cells) with ChAT expressing plasmid (Genecopoeia, EX‐T2064‐M10) (12 µg) was added to the cells using Lipofectamine 2000 (Invitrogen # 11668019) and Opti‐MEM (Gibco #31985070). Briefly, the plasmid was combined with 750 uL Opti‐MEM and another tube contained 18 uL of Lipofectamine 2000 and 732 uL of Opti‐MEM. Both tubes were vortexed and incubated at room temperature for 5 min. Then the tubes were combined and mixed by gently pipetting up and down. The mixture was incubated at room temperature for 20 min. After the incubation, the mixture was added to the cells. Cells transfected with empty vector were used as a control using the method described for ChAT plasmid. The ChAT protein generated had a C‐terminal histidine‐tag. Three days after transfection, the cells were collected into 1.25 ml of wash buffer (1% Triton X‐100, 50 mM Tris‐HCl, 500 mM NaCl, 20 mM imidazole, 2 mM beta‐mercaptoethanol). The cell lysates were centrifuged at 20,000 × *g* for 10 min in 4°C. Supernatant was combined with 200 uL of nickel (Ni‐NTA) beads (Invitrogen, 60‐0442) and incubated for 30 min at 4°C with rocking on a nutating rocker. The cell lysate and beads were added to a spin column and centrifuged at 500 × *g* for 1 min at 4°C. The columns were washed three times with 500 µL of 1% Triton wash buffer and centrifuged at 500 × *g* for 1 min at 4°C each time. The ChAT was eluted with 100 µL of 1% Triton elution buffer (1% Triton X‐100, 50 mM Tris‐HCl, 500 mM NaCl, 250 mM imidazole, 2 mM beta‐mercaptoethanol). The elution was repeated two more times. All eluates were combined. SDS‐polyacrylamide gel electrophoresis with a 10% gel was used to determine the concentration of recombinant ChAT protein. Increasing concentrations of BSA was added to different wells in the gel along with the eluates of the ChAT and vector wells. The gel was stained with EZBlue (Sigma, G1041) stain to visualize the bands. ChAT band was identified by size and absence from vector eluate. Image J and Graphpad Prism were used to quantify bands and estimate the concentration of ChAT (data not shown). The goal of quantification in the ELISAs was to maintain robust intra‐assay and inter‐assay consistency in measurement across different brain regions and cases. Measurement of absolute quantification is limited by potential difference in antibody binding to recombinant protein/synthetic peptide versus solubilized protein isolated from frozen tissue samples.

### 2.11 Lower limit of quantification

The lower limit of quantification (LLQ) was determined by the lowest point on the standard curve that had a %CV for the two absorbance readings lower than 15% and a percent recovery of 80%–120%.^48^ The LLQ was determined for all plates, and the LLQ determined for the majority of the plates was then applied for all the plates. Any sample below the LLQ was assigned a value of half of the LLQ.

## 3 RESULTS

### 3.1 Demographic and clinical information of participants

Table 1 summarizes the demographic and clinical information of the study participants. Table S1 provides additional clinical and pathological information for each participant, including dopaminergic and acetylcholinesterase inhibitor medications, Braak LB stage, and LB scores for substantia nigra and NBM. Age at death, PD duration, and all other listed variables did not significantly differ between control, PD without dementia (PD), and PD with dementia (LBD) participants (all p's > 0.05). The male/female ratio was higher, although non‐significant, in PD without dementia (PD) and PD with dementia (LBD), compared to controls (Ctrl).

### 3.2 ChAT immunostaining of cholinergic axons in cortex

To determine whether the antibodies utilized for the ChAT ELISA also detect ChAT protein in tissue sections prepared from frozen *post mortem* tissue samples, we used cryostat tissue sections prepared from control frozen *post mortem* cortex that were fixed for 15 min in 4% PFA after sectioning. Immunostaining with the goat anti‐ChAT antibody that was used as the capture antibody in the ELISA identified axons in cortex (Figure 1A, C), where ChAT protein appeared evenly distributed throughout the portions of axons visible in the sections. Similar results were observed for samples from three different control autopsy cases. We observed a similar pattern of immunostaining with a goat anti‐ChAT antibody that was previously utilized for IHC to assess cholinergic axon density in early Alzheimer disease^50^ (Figure 1B, D). In this prior study, the antibody showed more extensive staining of axons and varicosities in the cortex in tissue fixed in 4% PFA for 24–48 h and cryoprotected in graded sucrose concentrations (10%–30%) for several days prior to sectioning. The highly similar immunostaining patterns for these two antibodies in the frozen *post mortem* tissue sections as shown in Figure 1 support the specificity of the ELISA capture antibody for ChAT. We did not observe immunostaining with the mouse anti‐ChAT antibody that was used for detection in the ELISA.

**FIGURE 1:**
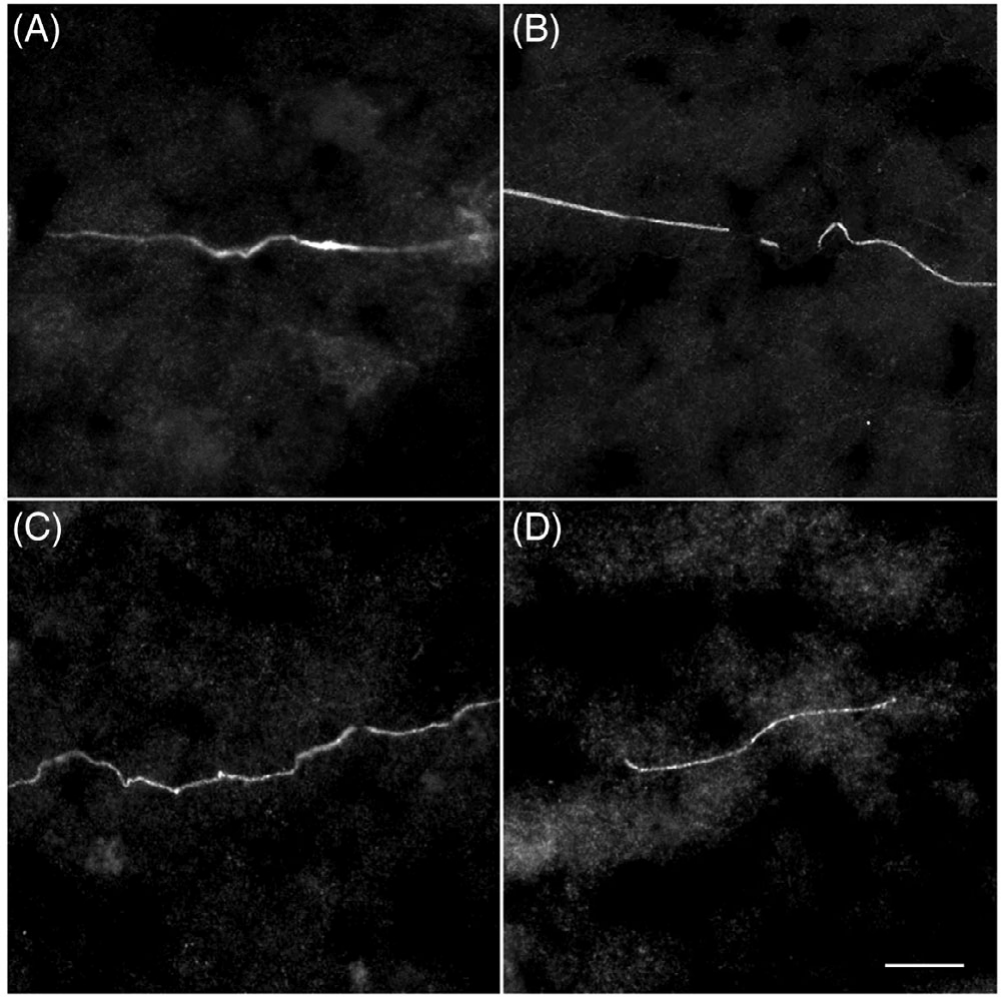
Immunofluorescence staining for ChAT in *post mortem* cortex samples from control cases. Frozen sections prepared from control *post mortem* tissue samples were immunostained with two anti‐ChAT antibodies, a goat anti‐ChAT antibody (R&D) that was also used as the capture antibody in the ELISA (A,C), and a goat anti‐ChAT antibody (Sigma) that was used in a previous study^63^ (B,D). Frozen sections were obtained from the frontal cortex of a vascular parkinsonism case (A,B), from the ACG of a dystonia case (C), and from the ACG of a cognitively normal control case (D). ChAT protein identified with both anti‐ChAT antibodies appeared evenly distributed throughout the portions of axons visible in the sections from the three cases, with some loss of continuity due to freezing artifact. Scale bar is 10 µm. ACG, anterior cingulate gyrus; ChAT, choline acetyltransferase; ELISA, enzyme‐linked immunosorbent assay.

### 3.3 Assay performance characteristics of the ELISA assays

We developed and validated a sandwich ELISA for ChAT, utilizing a goat anti‐ChAT antibody that was also used in the above immunostaining experiments for the capture antibody, and a mouse anti‐ChAT antibody as the detection antibody. We produced recombinant ChAT protein containing a C‐terminal six histidine tag by transient transfection of HEK293 cells with a human ChAT expression plasmid. Purified recombinant ChAT protein was used to optimize and calibrate the ELISA. Representative standard curves for the ELISA are shown in Figure S1. The ELISA LLQ for ChAT was 0.04 ng/ml, which corresponded to 0.62 µg/g wet weight tissue when adjusted for the sample dilution used in the ELISA. The percent coefficient of variation (%CV) for four inter‐plate controls was the most relevant measures of assay consistency between plates, which was 12%.

### 3.4 ChAT measurements in *post mortem* tissue samples

To analyze ChAT protein in frozen *post mortem* tissue samples, we isolated ChAT protein by sequential extraction and centrifugation. ELISA analysis indicated that ChAT protein was efficiently extracted in the initial soluble fractions obtained from a sequential extraction protocol utilizing buffers with increasing solubilization strength. We analyzed samples from eight brain regions: MFG, ACG, caudate, IPL, VAC, precuneus, hippocampus, and amygdala. These regions were chosen for the purpose of characterizing whether global cognitive impairment and specific cognitive phenotypes are related to measures of pathologic protein accumulation as well as projections from multiple subcortical nuclei including NBM, locus coeruleus, and dorsal raphe nuclei in PD. For this study, we used the sandwich ELISA to measure ChAT in samples from the PD, LBD, and control autopsy cases (Table S2).

### 3.5 Comparison of ChAT levels across PD, LBD, and control samples

PD and LBD cases had significantly lower ChAT levels than controls in most brain regions (Figure 2A‐C). In PD cases, ChAT levels were significantly lower than controls in the MFG, ACG, IPL, VAC, precuneus, and hippocampus, but not in the caudate and amygdala (Table 2A). In LBD cases, ChAT levels were significantly lower than controls in the MFG, ACG, IPL, VAC, precuneus, and hippocampus, but not in the caudate and amygdala (Table 2B). When PD and LBD cases were combined, ChAT levels remained significantly lower than controls in the MFG, ACG, IPL, VAC, precuneus, and hippocampus, while the caudate and amygdala were not significantly different (Table 2C). Comparisons between LBD and PD revealed that ChAT levels were significantly lower in LBD compared to PD in the IPL (while trends toward lower ChAT levels in LBD were observed in the MFG and precuneus) (Table 2D). Significant results remained after Holm–Bonferroni correction for multiple comparisons. There were large variations in ChAT levels in the caudate but the variation was similar among the three subgroups, which suggests it is not disease related.

**FIGURE 2:**
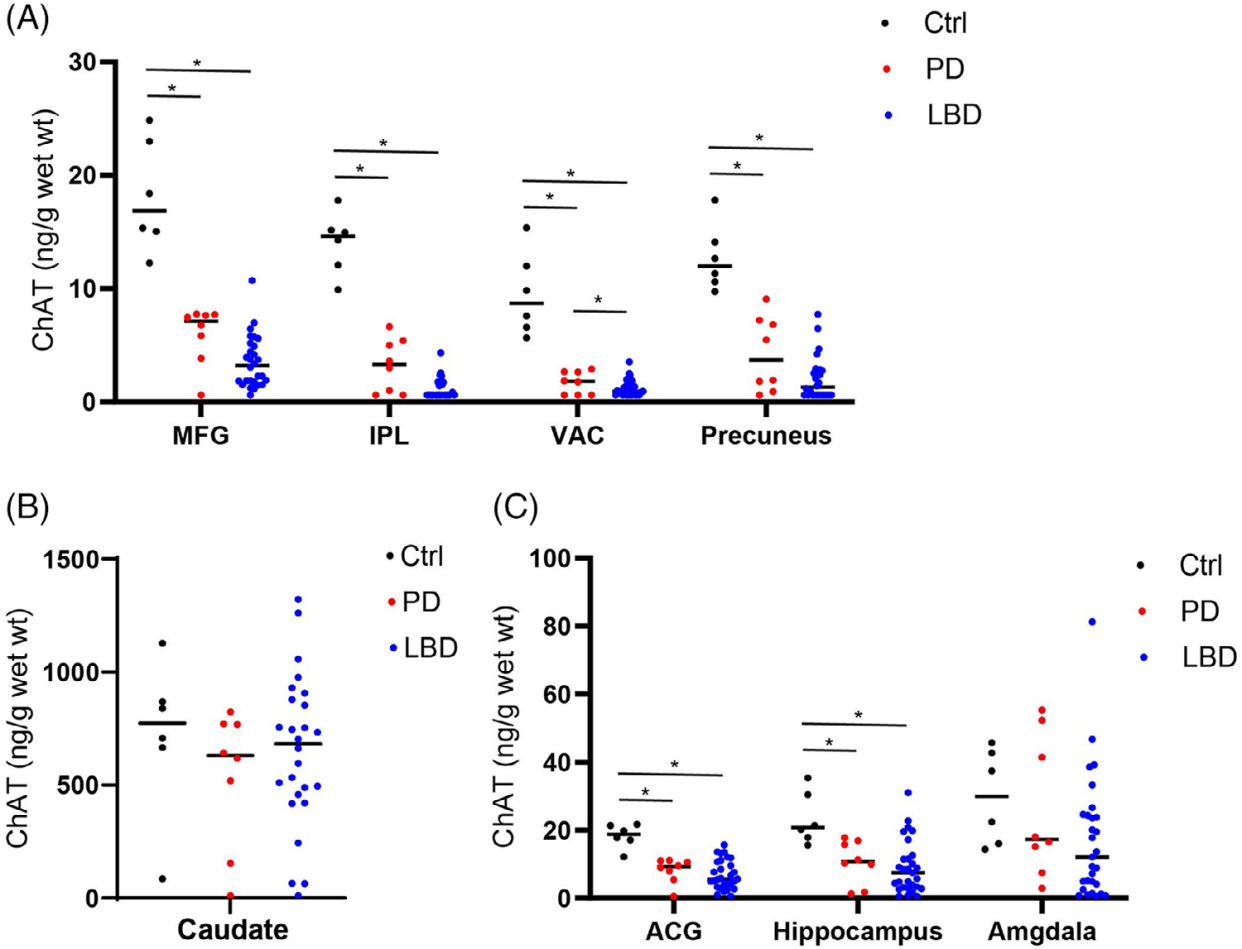
Regional distribution of ChAT in PD with dementia (LBD), PD without dementia, and control cases. Levels of ChAT measured by sandwich ELISA for PD with dementia (LBD) (blue circles), PD without dementia (red circles) and control (black circles) cases in (A) four neocortical brain regions (Mid‐Frontal Gyrus [MFG], Inferior Parietal Lobule [IPL] Visual Association cortex [VAC], Precuneus), (B) caudate, and (C) anterior cingulate gyrus (ACG), hippocampus and amygdala. ChAT levels were significantly higher in control cases compared to PD in six regions (MFG, ACG, IPL, VAC, precuneus, and hippocampus) and compared to LBD in the same six regions regions, as indicated by asterisks (*p* < 0.05, after correction for multiple comparisons). ChAT levels were significantly lower in LBD cases compared to PD without dementia cases in the IPL. The LLQ for the ELISA was 40 pg/ml or 0.62 ng/g wet wt tissue. Data were analyzed with the two‐tailed Mann–Whitney test using a significance level of 0.05 and corrected for multiple comparisons with the Holm–Bonferroni method.^52^ ChAT levels for the LBD cases were below the LLQ in IPL for 19 cases, in the VAC for nine cases and in the precuneus for 12 cases. There were also two and three cases for the PD below the LLQ in the regions of IPL and VAC, respectively. The regions of MFG and ACG had one case for both PD and LBD below the LLQ. The hippocampus had two LBD and amygdala had one LBD below LLQ. No cases were below LLQ for caudate. The highest value in amygdala was a significant outlier (Grubbs’ test). It did not affect the statistical significance of the subgroup comparisons. ACG, anterior cingulate gyrus; ChAT, choline acetyltransferase;ELISA, enzyme‐linked immunosorbent assay; IPL, inferior parietal lobule; LBD, Lewy body disease; LLQ, lower limit of quantification; MFG, middle frontal gyrus; PD, Parkinson's disease; VAC, visual association cortex.

**TABLE 2:** Statistics for subgroup comparisons for ChAT levels in each brain region and mean neocortical levels.

	Ctrl vs. PD					Ctrl vs. LBD			
A	*p*	*U*	*Z*	% of Ctrl	B	*p*	*U*	*Z*	% of Ctrl
MFG	0.0007	0	−3.1	42.2	MFG	<0.0001	0	−3.79	19.1
IPL	0.0007	0	−3.1	22.6	IPL	<0.0001	0	−3.81	4.2
VAC	0.0007	0	−3.1	20.7	VAC	<0.0001	0	−3.81	10.8
Precuneus	0.0007	0	−3.1	30.9	Precuneus	<0.0001	0	−3.79	10.9
Caudate	0.1812	13	−1.42	81.5	Caudate	0.5557	65	−0.63	88.2
ACG	0.0007	0	−3.1	49.3	ACG	<0.0001	4	−3.63	29.8
Hippocampus	0.0047	3	−2.71	51.9	Hippocampus	0.001	17	−3.06	35.7
Amygdala	0.7546	21	−0.39	57.6	Amygdala	0.0617	44	−1.88	40.4
Neocortical	0.0007	0	−3.1	33.3	Neocortical	<0.0001	0	−3.78	11.3

	Ctrl vs. PD/LBD					PD vs. LBD			
C	*p*	*U*	*Z*	% of Ctrl	D	*p*	*U*	*Z*	% of PD
MFG	<0.0001	0	−3.88	22.6	MFG	0.0122	48	−2.45	45.3
IPL	<0.0001	0	−3.89	4.2	IPL	0.0044	50	−2.43	18.6
VAC	<0.0001	0	−3.89	11.4	VAC	0.2951	88	−1.05	51.8
Precuneus	<0.0001	0	−3.88	14.6	Precuneus	0.0747	66	−1.75	35.4
Caudate	0.3802	78	−0.91	84.4	Caudate	0.6239	92	−0.51	108.2
ACG	<0.0001	4	−3.75	35.8	ACG	0.3968	93	−0.87	60.5
Hippocampus	0.0005	20	−3.19	41.2	Hippocampus	0.4754	96	−0.74	68.7
Amygdala	0.1124	65	−1.61	50.6	Amygdala	0.2075	81	−1.29	70.1
Neocortical	<0.0001	0	−3.87	13.1	Neocortical	0.0505	58	−1.96	34

*Note*: Mann–Whitney *P*, *U*, and *Z* values and % of control (A,B,C) or PD (D) are listed for each comparison.

Abbreviations: ACG, anterior cingulate gyrus; ChAT, choline acetyltransferase; IPL, inferior parietal lobule; LBD, Lewy body disease; MFG, middle frontal gyrus; PD, Parkinson's disease; VAC, visual association cortex.

### 3.6 Relationships between ChAT levels and cognitive impairment

We hypothesized that measures of global cognitive impairment, including a diagnosis of dementia based on the CDR global score, would relate primarily to deficits in cholinergic innervation across neocortical regions. We also hypothesized that impairment in specific cognitive domains would relate to cholinergic deficits in individual regions but determined that examination of these relationships will require higher number of samples for increased statistical power; thus, analyses here focus on global metrics of cognitive function. Mean levels of ChAT measured for the four neocortical regions (MFG, IPL, VAC, and precuneus) were 33% of Control levels in PD cases and 11% of Control levels in LBD cases. They trended lower in LBD cases compared to PD cases, but the difference was not significant (Figure 3). First, we tested the relationship of ChAT with demographics, clinical characteristics, and neuropathology. There were no significant correlations between mean neocortical ChAT levels and disease duration, age at PD onset, age at death, years of education, or pathology (Braak amyloid, Braak NFT, or Braak LB stages), *post mortem* interval, nor differences based on sex (data not shown; all *p* > 0.05). There were also no significant correlations between ChAT and insoluble total asyn, phospho‐asyn, Aβ, total tau and phospho‐tau previously measured for the same tissue samples from 15 of the autopsy cases (data not shown; all *p* > 0.05) [^30^].

**FIGURE 3:**
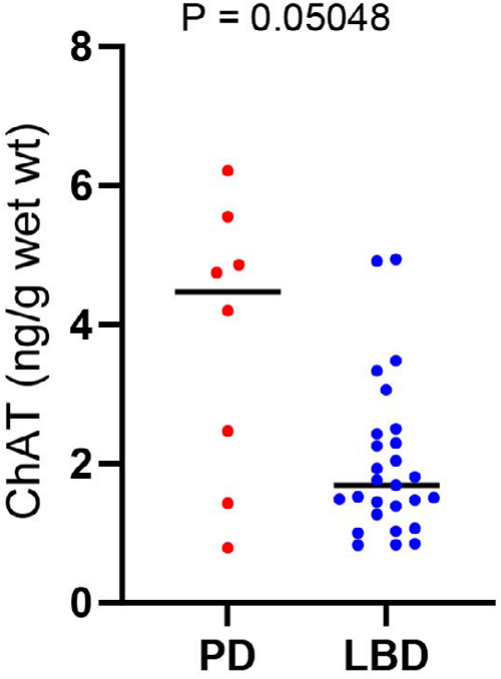
Comparison of mean neocortical ChAT in PD with dementia (LBD) and PD without dementia. The mean levels of ChAT for the neocortical regions of MFG, IPL, VAC and precuneus were compared between PD with dementia (LBD) (blue circles) and PD without dementia (red circles) cases. Mean neocortical ChAT levels for LBD were trending lower (*p* = 0.05) than PD without dementia cases. ChAT, choline acetyltransferase; IPL, inferior parietal lobule; LBD, Lewy body disease; LBD, Lewy body disease; MFG, middle frontal gyrus; PD, Parkinson's disease; VAC, visual association cortex.

Next, we utilized Pearson and partial Pearson correlations to analyze the relationship between measures of global cognitive function (i.e., CDR sum of boxes, MMSE, Cog‐Z score) and mean neocortical ChAT levels for all PD and LBD cases (Figure 4).

**FIGURE 4:**
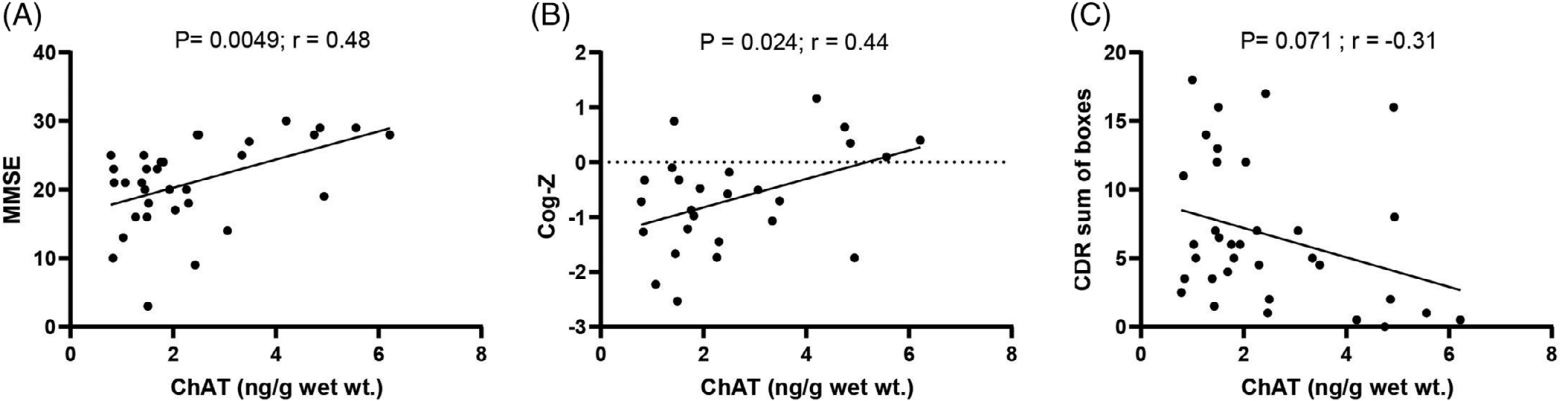
Correlations between measures of cognitive function and neocortical ChAT levels in all PD/LBD cases. We used Pearson and partial Pearson correlations to analyze cognitive measures (MMSE and Cog‐Z, lower score indicates more cognitive impairment; CDR sum of boxes, higher score indicates more cognitive impairment) in relation to mean neocortical ChAT. (A) There was a significant positive correlation between MMSE and mean neocortical ChAT (Pearson: r = 0.48, p = 0.0049). (B) There was also a significant positive correlation between Cog‐Z and mean neocortical ChAT (Pearson: r = 0.44, p = 0.024). (C) There was a trending inverse correlation between CDR sum of boxes and mean neocortical ChAT (Pearson: r = −0.31, p = 0.071). Partial Pearson correlations, controlling for time interval between data collection and death as well as education, further showed positive associations between neocortical ChAT and MMSE (r = 0.50, p = 0.005) and Cog‐Z (r = 0.58, p = 0.005), and a trending inverse relationship with CDR sum of boxes (r = −0.33, p = 0.066). Scatter plot shows raw scores with linear regression lines. ChAT, choline acetyltransferase; CDR, Clinical Dementia Rating; Cog‐Z, global cognition z‐score; MFG, middle frontal gyrus; IPL, inferior parietal lobule; LBD, Lewy body disease; MMSE, Mini‐Mental State Examination; PD, Parkinson's disease; VAC, visual association cortex.

Pearson correlations revealed that neocortical ChAT concentration was positively related to global cognitive function as measured by MMSE (*r* = 0.48, *p* = 0.005) and Cog‐Z scores (*r* = 0.44, *p* = 0.024), with lower ChAT levels related to worse cognition, while cognitive impairment as indicated by higher CDR sum of boxes showed a trending inverse correlation (*r* = −0.31, *p* = 0.071) with lower ChAT levels. Partial Pearson correlations were conducted controlling for time interval between data collection and death, as well as education. Notably, the mean time interval between CDR collection and death was 1.7 ± 1.6 years, between MMSE collection and death was 1.2 ± 1.5 years, and between Cog‐Z collection and death was 1.9 ± 1.6 years. These partial correlations further showed positive associations between neocortical ChAT and MMSE (*r* = 0.50, *p* = 0.005), and Cog‐Z (*r* = 0.58, *p* = 0.005), and a trending inverse relationship with CDR sum of boxes (*r* = −0.33, *p* = 0.066).

## 4 DISCUSSION

We report the results of a new approach to analyze the loss of cholinergic axon projections in *post mortem* tissue from PD with and without dementia. Our analysis relies on extraction of soluble proteins from frozen *post mortem* tissue samples, followed by quantification of ChAT levels with a sandwich ELISA. We applied these methods across eight brain regions in 29 PD with dementia, eight PD without dementia, and six control cases. ChAT levels were significantly lower than controls in PD cases across six regions (MFG, ACG, IPL, VAC, precuneus, hippocampus) but not in the caudate or amygdala. In LBD cases, all regions except the caudate and amygdala also showed significant reductions. These results indicate significant loss of cholinergic projections from NBM to cortical regions in PD and LBD, as well as significant loss of cholinergic projections from medial septum and the vertical limb of the diagonal band of Broca to hippocampus. Compared to PD, LBD cases had significantly lower ChAT in the IPL with trends toward lower levels in the MFG and precuneus. Importantly, neocortical ChAT levels for PD and LBD cases were significantly related to measures of cognitive impairment. Specifically, lower mean neocortical ChAT levels were related to worse MMSE and global Cog‐Z score, and showed a trend‐level association with worse CDR sum of boxes. This indicates that lower levels of cholinergic innervation in neocortical areas are linked to greater cognitive impairment. Together, these results strongly suggest that deficits of cholinergic innervation in neocortical regions contribute to cognitive impairment in PD.

Our ELISA data expands upon previous studies utilizing radiolabeled enzyme activity measurements in *post mortem* tissue extracts. Previous studies have shown that ChAT enzyme activity in frontal cortex, temporal cortex, and hippocampus from *post mortem* PD brain tissue was decreased up to 80% compared to controls, and PD patients with dementia had lower ChAT activity compared to patients without dementia, utilizing a total of 8–16 PD autopsy cases.^23^, ^26^, ^27^, ^28^, ^51^ Our results significantly expand the analysis of ChAT in *post mortem* tissue by utilizing a sandwich ELISA approach to quantify ChAT levels in multiple cortical regions from a large number of cases, and by identifying correlations between ChAT levels and cognitive impairment. Cholinergic axons in cortical regions have not been analyzed by immunostaining in *post mortem* PD tissue with methods similar to those used to analyze loss of cholinergic axons in Alzheimer's disease (AD) tissue and loss of cholinergic terminals in the hippocampal CA2 region in PD.^52^ This type of analysis could further characterize the reduction in ChAT protein by determining whether it reflects a substantial loss in the density of axons identified by ChAT immunostaining or whether the number of ChAT axons is preserved but with lower ChAT levels. However, it is also possible that a substantial percentage of axons with reduced ChAT concentration would not be detected by immunostaining if levels are below the sensitivity limit of an immunostaining approach. Importantly, whether the ELISA measurements of reduced ChAT levels in tissue samples arise from decreased axon density or decreased ChAT concentrations in axons, they indicate a substantial loss of cholinergic function related to a decreased capacity for synthesis and release of acetylcholine. Thus, this new quantitative approach to measure loss of cholinergic function can be utilized to further characterize the role of cholinergic neuron degeneration in global cognitive impairment as well as impairment in specific cognitive domains in PD. It can also be utilized in future studies of AD where analysis of cortical regions in *post mortem* tissue samples indicate that ChAT activity is not decreased relative to controls in mild cognitive impairment (MCI) and mild AD, but is decreased in later stage AD, and that ChAT activity is elevated in frontal cortex and hippocampus for MCI compared to controls,^53^ a pattern in relation to disease progression that differs from our measurements in PD and LBD. Selective loss of cholinergic neurons in the ventral striatum has also been observed in AD, which may not be sufficiently represented in our caudate samples for comparison with PD/LBD.

Loss of cholinergic projections to cortex in PD have also been examined through analysis of other protein markers specific to cholinergic neurons that are less directly related to acetycholine production but may correlate with the density of cholinergic terminals and their function. In a proteomic study utilizing mass spectrometry measurements of proteins isolated from *post mortem* frontal cortex, the choline transporter protein, which is specific to cholinergic neurons, was lower in PD samples compared to controls.^54^ In PET imaging studies with a tracer for VAChT, which is present only on presynaptic cholinergic nerve terminals, PD participants have demonstrated lower signal compared to controls in nucleus basalis of Meynert and multiple cortical regions.^9^, ^23^, ^55^, ^56^, ^57^, ^58^, ^59^ In addition, PD participants with dementia revealed lower signal with this cholinergic tracer compared to those without dementia.^5^, ^23^, ^51^, ^55^ PET signal for VAChT in cortical regions for PD with dementia was approximately 60% of levels observed in controls, in contrast to the more substantial reductions in ChAT levels (11% of control levels for neocortical regions) observed in our ELISA study and also in previous studies measuring ChAT enzyme activity. It is not clear whether this difference is explained by more advanced disease progression in the autopsy cases or whether ChAT levels are reduced to a greater extent than vAChT levels. Clinical studies demonstrate that drugs that inhibit acetylcholinesterase (AChE) produce partial improvement in cognitive function for participants with PD.^17^ Some imaging studies utilizing tracers for AChE have not shown deficits in basal forebrain or cortical regions.^18^, ^60^ However, since AChE is localized to both presynaptic and postsynaptic neurons, AChE measurements may underestimate the reduction in cholinergic neurons compared to VAChT tracers, *post mortem* ChAT measurements, or cell counts.^61^

There are several limitations to understanding the cholinergic system's role in the development of dementia in PD. This study is limited to patients who donated their brains through our MDC and may underrepresent individuals who present with dementia first or early in the disease, since these patients are more likely to be seen at centers specializing in cognitive impairment. Currently, we only report one measurement for cholinergic neurons. There may not be a simple linear relationship between cholinergic function and the level of a single marker, given the complex roles of enzymes and transporters in synaptic function and the potential for disease‐related compensatory changes in protein expression levels.^50^ Therefore, the assessment of multiple markers in parallel may provide a more complete picture for both *post mortem* tissue studies, as well as in vivo imaging studies. For example, development of a ChAT PET tracer could complement results obtained with VAChT PET tracers and structural MRIs. Similarly, measurements of VAChT and the choline transporter (CHT1)^54^ could complement results of ChAT analysis in tissue samples, a goal for future assay development efforts. These studies would also enable comparison of how these different markers change in relation to disease progression and cognitive impairment. Our analysis approach is best applied to brain regions that are large enough to enable dissection from 1 cm thick coronal slices of frozen *post mortem* brain tissue, which is challenging with small subcortical nuclei. Although ELISAs enable parallel measurements with large numbers of tissue extracts, the time for tissue dissection and extraction limits throughput to some extent. Finally, *post mortem* tissue analysis captures only a single timepoint, often later in the disease course, and therefore does not enable longitudinal assessment of progression. An advantage of *post mortem* tissue analysis, however, is the ability to measure multiple analytes from the same samples, enabling investigation of multiple processes, such as loss of cholinergic innervation, loss of projections from other subcortical nuclei, pathologic protein accumulation, and synaptic terminal loss, as well as their interrelationships.

Additional studies are needed to better understand the relationship between the cholinergic system and dementia, including the specific cognitive phenotypes associated with PD. For example, analysis with a larger sample size may identify relationships between loss of cholinergic innervation in individual brain regions to deficits in specific cognitive domains. An advantage of this ELISA approach, in which inter‐assay controls are included on each assay plate to assess the precision of ChAT measurements, is that the current data set can be expanded by analyzing additional autopsy cases and samples with the same ELISA protocol, The inclusion of more cases without dementia would be particularly helpful for future *post mortem* tissue studies, though this remains challenging given the relatively low frequency of these cases at autopsy among cohorts of movement disorders, dementia, and aging studies. Analysis of additional markers for cholinergic neurons would also be beneficial to complement the ChAT measurements since different markers may have different relationships to cholinergic function. Finally, dementia in PD is also related to accumulation of pathologic asyn in cortical regions, as observed by studies utilizing either IHC^62^, ^63^, ^64^, ^65^ or ELISA analysis of aggregated asyn protein.^30^ Pathologic asyn accumulation in the cortex is likely associated with loss of function and neurodegenerative changes in cortical neurons. Future studies combining cholinergic measures with additional assays to measure neurodegeneration, as well as pathologic asyn accumulation in the cortex, may be particularly valuable in understanding substrates for cognitive impairment in PD.

## 5 CONCLUSIONS

These results support a role for the loss of cholinergic axon projections to cortex in the development of cognitive impairment in PD.

## AUTHOR CONTRIBUTIONS

*Study concept and organization*: P.K., J.P., and M.C. *Execution* and *Acquisition of data*: R.M., J.O., S.L., H.H., E.F., R.P. *Analysis and interpretation of data*: R.M., R.P., M.C., J.P., P.K., L.R. *Drafting of the manuscript*: R.M. and P.K. *Critical revision of the manuscript for important intellectual content*: All authors. *Statistical analysis*: R.M., P.K., L.R. and M.C. *Obtained funding*: P.K., M.C. and J.P.

## CONFLICT OF INTEREST STATEMENT

The authors declare that they have no competing interests for this study. Author disclosures are available in the supporting information. Author disclosures are available in the Supporting Information.

## CONSENT STATEMENT

This research was conducted in accordance with the Declaration of Helsinki. The Human Research Protection Office at Washington University in Saint Louis approved this study. Written informed consent to perform a brain autopsy was obtained from all participants. After death, the immediate next‐of‐kin were contacted and confirmed consent for brain removal and retention of brain tissue for research purposes.

## Supporting information



Supporting Information

Supporting Information

Supporting Information

Supporting Information

## Data Availability

The data that supports the findings of this study are available in the supplementary material of this article.
