# Supplementary material for: Cognitive impairment in Parkinson disease correlates with loss of cholinergic axon projections to cortex in an autopsy study
Source: Alzheimers Dement. 2026 Oct 6;22(10):e71868. doi: 10.1002/alz.71868 (PMC13641098; doi:10.1002/alz.71868)
Supplement: Supplementary file 1 [file ALZ-22-e71868-s002.pdf]

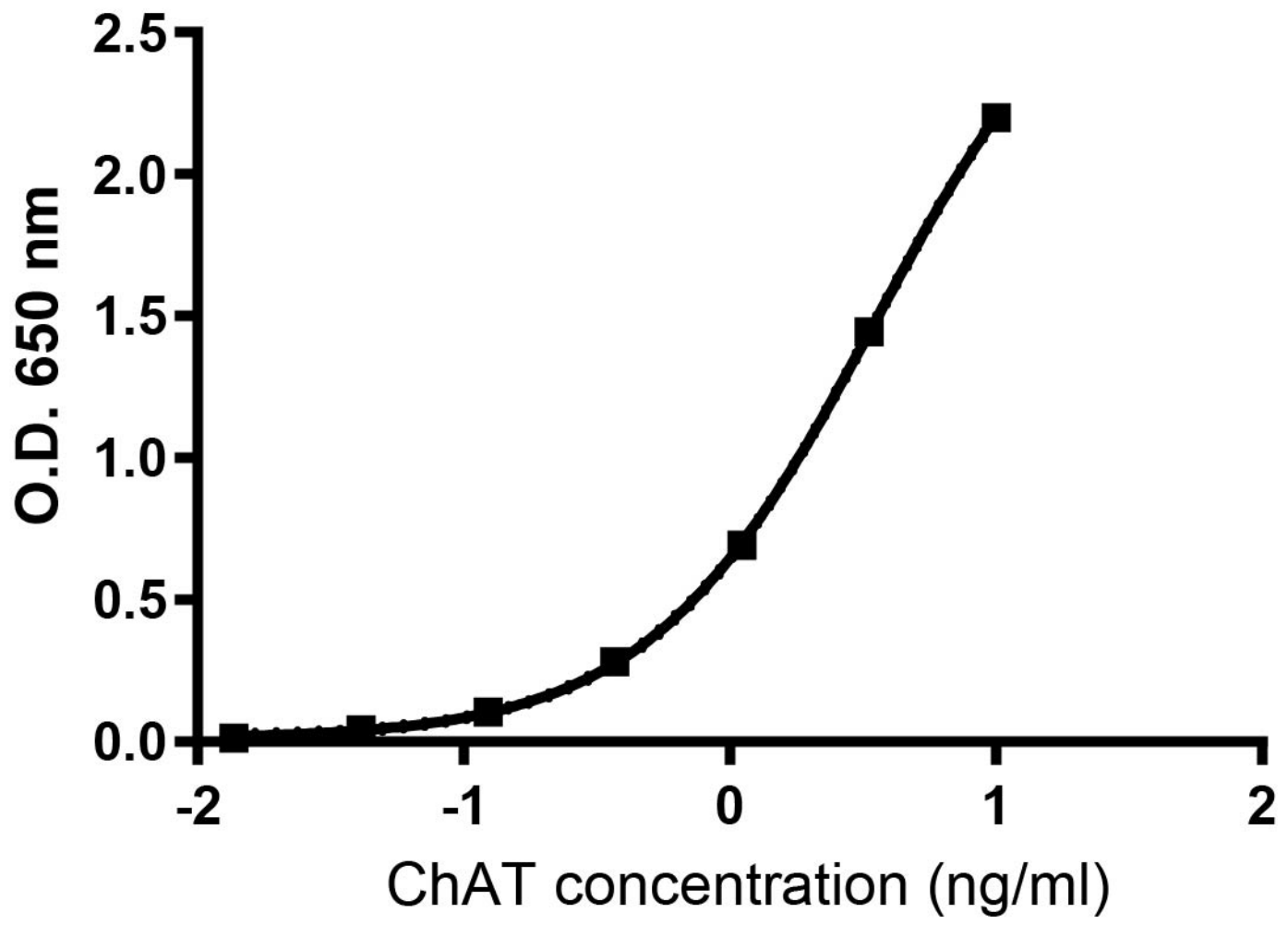

Supplementary Figure 1: Representative standard curve for ChAT ELISA
The standard curve was run in duplicate wells. Full-length standard curves are shown. Concentrations are graphed in log scale. The mean and error of duplicates are graphed.
